# *Citrus aurantium* L. dry extracts promote *C/ebpβ* expression and improve adipocyte differentiation in 3T3-L1 cells

**DOI:** 10.1371/journal.pone.0193704

**Published:** 2018-03-29

**Authors:** Gregory Alexander Raciti, Francesca Fiory, Michele Campitelli, Antonella Desiderio, Rosa Spinelli, Michele Longo, Cecilia Nigro, Giacomo Pepe, Eduardo Sommella, Pietro Campiglia, Pietro Formisano, Francesco Beguinot, Claudia Miele

**Affiliations:** 1 URT of the Institute of Experimental Endocrinology and Oncology “G. Salvatore”, National Council of Research, Naples, Italy; 2 Department of Translational Medical Sciences, “Federico II” University of Naples, Naples, Italy; 3 Department of Pharmacy, School of Pharmacy, University of Salerno University of Salerno, Fisciano, Italy; 4 European Biomedical Research Institute of Salerno, Salerno, Italy; Tokyo University of Agriculture, JAPAN

## Abstract

Metabolic and/or endocrine dysfunction of the white adipose tissue (WAT) contribute to the development of metabolic disorders, such as Type 2 Diabetes (T2D). Therefore, the identification of products able to improve adipose tissue function represents a valuable strategy for the prevention and/or treatment of T2D. In the current study, we investigated the potential effects of dry extracts obtained from *Citrus aurantium* L. fruit juice (*CA*de) on the regulation of 3T3-L1 cells adipocyte differentiation and function *in vitro*. We found that *CA*de enhances terminal adipocyte differentiation of 3T3-L1 cells raising the expression of *CCAAT/enhancer binding protein beta* (*C/Ebpβ*), *peroxisome proliferator activated receptor gamma* (*Pparγ*), *glucose transporter type 4* (*Glut4*) and *fatty acid binding protein 4* (*Fabp4*). *CA*de improves insulin-induced glucose uptake of 3T3-L1 adipocytes, as well. A focused analysis of the phases occurring in the pre-adipocytes differentiation to mature adipocytes furthermore revealed that *CA*de promotes the early differentiation stage by up-regulating *C/ebpβ* expression at 2, 4 and 8 h post the adipogenic induction and anticipating the 3T3-L1 cell cycle entry and progression during mitotic clonal expansion (MCE). These findings provide evidence that the exposure to *CA*de enhances *in vitro* fat cell differentiation of pre-adipocytes and functional capacity of mature adipocytes, and pave the way to the development of products derived from *Citrus aurantium* L. fruit juice, which may improve WAT functional capacity and may be effective for the prevention and/or treatment of T2D.

## Introduction

A recent report on diabetes by the World Health Organization estimates that the number of adults living with diabetes has almost quadrupled since 1980 passing from 108 million to 422 million cases in 2014 [1]. This dramatic rise is largely due to Type 2 Diabetes (T2D), and is the result also of metabolic and/or endocrine dysfunction occurring at the white adipose tissue (WAT) [2–4]. Metabolic disturbances, alterations in adipokine secretion, and low grade inflammation of the WAT have indeed deleterious effects on insulin sensitivity and lead to both locally and whole-body insulin resistance [5–7]. Therefore, the ways to manage T2D and/or delay the onset of its complications pass also through the generation of compounds, which improve the functional capacity of WAT.

Nutraceuticals are nutritional products derived from plants and food sources with health or medical benefits. Recent evidence from human and animal studies nowadays strengthens their use as complementary strategy in support to the pharmacological treatment of several diseases, including T2D [8–13]. Some of them are, indeed, being used as good co-adjuvants along with the balanced diets and with the currently used drugs for the management of the blood glycaemia and for the prevention and treatment of T2D [8, 9, 14–23]. E.g., oral administration of the high molecular weight *Gymnema sylvestre* R. Br. leaf extract, Om Santal Adivasi, increases circulating serum insulin and reduces both fasting and post-prandial blood glucose in humans [19]. Also, oral assumption of hydroalcoholic extracts of *Trigonella foenum-graecum* L. seeds improves glycemic control and decreases insulin resistance in T2 diabetics [23].

*Citrus aurantium* L., also known as “bitter orange”, is a common plant present in the Mediterranean basin, whose health properties have been described since the time of the ancient Greeks and Romans [24]. *Citrus aurantium* L., indeed, contains several bioactive compounds, including alkaloids, flavonoids, and polyphenols [25–28], and its nutraceutical activities are supported by several scientific and clinical studies [27–29]. E.g., extracts obtained from the immature fruits of *Citrus aurantium* L. are commonly used in weight management due to effects on thermogenesis regulation [28]. Also, specific bioactive components present in *Citrus aurantium* L., such as the alkaloid p-synephrine and its metabolite p-octopamine, exhibit sympathomimetic actions on the α- and β-adrenergic receptors thus modulating lipolysis of adipocytes [28, 30]. However, to date, due to substantial qualitative and quantitative differences in the composition among *Citrus aurantium* L. extract preparations, several studies conducted both at pre-clinical and clinical level have reported conflicting findings on the effective role of *Citrus aurantium* L. extracts as thermogenic agents [27, 28]. Furthermore, the effects of *Citrus aurantium* L. on adipose function have not been yet fully understood. The main criteria to investigate underlying mechanisms by which nutraceuticals may improve metabolic health targeting adipose tissue are the regulation of pre-adipocyte commitment and differentiation to mature adipocytes and the modulation of adipocyte glucose and fat metabolism [31–33]. Adipocytes are indeed the primary components of the adipose tissue and play a critical role in the regulation of adipose tissue energy homeostasis and endocrine function [5–7, 34–37].

Here, in an effort to disclose the nutraceutical properties of bioactive components present in *Citrus aurantium* L. on adipocyte function, we have used a preparation of *Citrus aurantium* L. dry extracts (*CA*de) obtained from its fruit juice and we have investigated its effects *in vitro* in 3T3-L1 pre-adipocytes.

## Materials and methods

### *Citrus aurantium* L. dry extract (*CA*de) preparation

Dry extracts from *Citrus aurantium* L. (*CA*de) fruit juice were obtained as previously described [38]. Briefly, *Citrus aurantium* L. fruit juice was provided by the company “Agrumaria Corleone” (Palermo, Italy) which used fruits harvested from *Citrus aurantium* L. plants cultivated in Eastern Sicily, Italy. In order to remove fibers, 100 mL of hand squeezed juice were centrifuged at 12000 rpm for 15 min at 25 °C, then lyophilized for 24 h by setting the condenser temperature at -52 °C and the vacuum value at 0.100 mBar. The powder was extracted with MeOH and the procedure was repeated three times for the complete recovery of polyphenolic compounds. The extract was filtered through 0.45 μm nylon membrane (Merck Millipore, Billerica, MA), evaporated under vacuum to dryness, and stored at 4°C until used. The lyophilized dried extracts were then re-hydrated with distilled H_2_O to a final concentration of 10 mg/ml. Treatments with *CA*de were made to concentrations and at time indicated in the following paragraphs of the section “Materials and Methods”. A qualitative and quantitative analysis of compounds in *CA*de was also performed through advanced processes of extraction and refining. Details are reported in Materials and Methods A and Table A in S1 File.

### Cell culture, adipocyte differentiation and treatments

3T3-L1 cells were obtained from the American Type Culture Collection (Manassas, VA) and cultured in pre-adipocyte expansion medium containing Dulbecco’s modified Eagle’s medium (DMEM, 4.5 g/L glucose) supplemented with 10% fetal calf serum (FCS, Thermo Fisher Scientific, Waltham, MA), 100 U/ml penicillin, and 100 mg/ml streptomycin, at 37 °C in a humidified atmosphere of 5% CO_2_ [39]. Media and antibiotics for cell culture were from Lonza (Walkersville, MD). 3T3-L1 cells were differentiated as previously described [40]. In details, to induce differentiation, 3T3-L1 pre-adipocytes were seeded in a 6-well culture plate at a density of 8.0 x 10^4^ cells per well. Two days after confluence (Day 0, D0), the pre-adipocyte expansion medium (DMEM—10% FCS) was removed and adipocyte differentiation was initiated by culturing growth-arrested 3T3-L1 pre-adipocytes for 48 h with the differentiation medium (MDI) containing DMEM (4.5 g/L glucose)—10% fetal bovine serum (FBS, Thermo Fisher Scientific) supplemented with 3-isobutyl-1-methylxanthine (0.5 mM, Sigma Aldrich, St Louis, MO), dexamethasone (1 μM, Sigma Aldrich) and insulin (1 μg/ml, Sigma Aldrich). Starting from day 2 (D2), cells were cultured in adipocyte maintenance medium containing DMEM (4.5 g/L glucose)—10% FBS supplemented with 1 μg/ml insulin, and medium was changed every 48 h until day 8 (D8). *CA*de (100 μg/ml) was added in every replacement except on experiments where it was added only from D0 to D2 or from D2 to D8. For the experiments with specific compounds, 3T3-L1 pre-adipocytes were seeded in a 6-well culture plate at a density of 8.0 x 10^4^ cells per well. Two days after confluence, growth-arrested 3T3-L1 pre-adipocytes were cultured in MDI for 2, 4 and 8 h in the presence of 6.7 μg/ml narirutin or 3.9 μg/ml hesperidin or 5.5 μg/ml vicenin-2. Compounds were from Extrasynthese (Genay, France).

### Sulforhodamine B (SRB) assay

SRB assay was performed as described [41]. The method was optimized for the toxicity screening of *CA*de in a 96-well plate. After 24 h of treatment, cells were fixed with 50% trichloroacetic acid at 4°C (100 μl/well, final concentration 10%) for 1 h, and then stained with 0.4% SRB (Sigma-Aldrich) dissolved in 1% acetic acid (50 μl/well) for 30 min. Excess of dye was removed by washing with l% (vol/vol) acetic acid. Plates were air-dried and protein-bound dye was solubilized in 100 mM Tris base solution. Optical density was determined at 490 nm, using a microplate reader.

### Triglyceride (TG) quantification assay

The cellular concentration of TG was determined according to previously reported method with few modifications [42]. Briefly, cells were washed twice with PBS 1X, scraped into PBS 1X and lysed by sonication. Cellular lysates were divided in 2 aliquots. One aliquot was assayed for total TG content measurement using a TG assay kit from Sigma-Aldrich. The other one was used for DNA quantitation using the AllPrep DNA/RNA/miRNA Universal kit (Qiagen, Hilden, Germany). For each sample, total TG content was therefore normalized to DNA concentration and results were expressed as μg TG per μg DNA.

### Oil-Red O staining

Oil-Red O staining was performed as described [43]. At D8, mature 3T3-L1 adipocytes were fixed with 10% formaldehyde for 5 min at room temperature and washed with PBS 1X and then dried completely. Fixed cells were stained with Oil-Red O (Sigma-Aldrich) in 60% isopropyl alcohol solution for 30 min at room temperature. Intracellular lipid content was quantified by dissolving Oil-Red O stain with 100% isopropyl alcohol and the optical density was measured at 490 nm by a spectrophotometer.

### 2-Deoxy glucose (2-DG) uptake

2-DG uptake was performed as previously described [44]. In details, 3T3-L1 cells differentiated for 8 days in mature adipocytes treated or not with *CA*de (100 μg/ml) from D0 to D8, or from D0 to D2 or from D2 to D8, were starved for 24 h in DMEM supplemented with 0.1% BSA. Adipocytes were then washed twice with KRH buffer (HEPES 50 mM, NaCl 137 mM, KCl 4.7 mM, MgSO_4_ 1.3 mM, CaCl_2_ 1.85 mM, BSA 0.1%) and stimulated or not with insulin (100 nmol/L) for 30 min. Glucose uptake was determined by the addition of 2-DG mix containing 1 mM of cold 2-DG and 0.25 μCi of 2-[^14^C]-DG for 5 min. Cells were then washed with KRH buffer and lysed with NaOH 0.05 M. The 2-DG uptake was quantified by liquid scintillation counting and normalized for protein content by Bradford protein assay.

### qPCR

RNA was isolated using AllPrep DNA/RNA/miRNA Universal kit (Qiagen). cDNA synthesis and qPCR were performed as described [45]. Primer sequences used in qPCR: *Cyclophilin*: F, 5’-gcaagcatgtggtctttggg-3’; R, 5’-gggtaaaatgcccgcaagtc-3’; *C/ebpα*: F, 5’-cgacttctacgaggtggagc-3’; R, 5’-tcgatgtaggcgctgatgtc-3’; *C/ebpβ*: F, 5’-cgcccgccgcctttagac-3’; R, 5’-cgctcgtgctcgccaatgg-3’; *C/ebpδ*: F, 5’-atcgctgcagcttcctatgt-3’; R, 5’-agtcatgctttcccgtgttc-3’; *Pparγ*: F, 5’-ggaagccctttggtgactttatgg-3’; R, 5’-gcagcaggttgtcttggatgtc-3’; *Glut4*; F, 5’-cctggaatgctgtctctgg-3’; R, 5’-tggctctgtcttaatgttgatg-3’; *Fabp-4*: F, 5’-aatcaccgcagacgacag-3’; R, 5’-acgcctttcataacacattcc-3’.

### Western blot (WB) analysis

WB analysis were performed as previously described [46]. Briefly, 3T3-L1 cells were harvested and washed in PBS 1X, and then lysed in Lysis Buffer containing 20 mM Tris-HCl, pH7.5, 150 mM NaCl, 5 mM EDTA, 1% NP40, 5 μg/ml leupeptin, 5 μg/ml aprotinin, 10 μM PMSF. Samples were incubated on ice 30 min after the addition of Lysis Buffer and then cell lysates were clarified by centrifugation at 15,000 g for 10 min at 4°C. The protein concentration of the cell lysate was determined using the Coomassie blue protein assay (Bio-Rad Laboratories, Hercules, CA). Protein lysates were then analyzed by SDS-PAGE, transferred to a PVDF membrane and subjected to WB analysis. Membranes were firstly probed with antibodies to phospho-CREB Ser133 (1B6; #9196) and CREB (48H2; #9197) from Cell Signaling Technology (Danvers, MA) and to β-Actin (I-19; sc-1616) from Santa Cruz Biotechnology Inc (Dallas, TX) and then probed with secondary mouse or rabbit antibodies (Bio-Rad Laboratories) before detection of the signal with ECL plus (GE Healthcare, Chicago, IL).

### Chromatin immunoprecipitation (ChIP)

ChIP assays was performed as described [40]. For ChIP assay, sonicated chromatin was immune-precipitated with the anti-CREB antibody (48H2; #9197; Cell Signaling Technology), and anti-rabbit IgG from Sigma-Aldrich. Relative protein binding to the *C/ebpβ* gene was evaluated on recovered DNA by qPCR. Primers used are the following: CREB bs: F, 5’-gccctctcgcgctc-3’; R, 5’-ggctccgctgcgtc-3’ Samples were normalized to their respective input using the 2^-ΔCT^ method.

### Cell growth and flow cytometry analysis

Cell growth analysis was performed as previously described [47]. Briefly, the mouse embryonic 3T3-L1 pre-adipocytes and the mouse embryonic fibroblasts NIH-3T3 cells were seeded in 6-well culture plates at a density of 8.0 x 10^4^ cells per well. The day after (day 0), 3T3-L1 pre-adipocytes were cultured in complete medium for the following 72 h in the presence or absence of *CA*de (100 μg/ml). Cell growth was analyzed by counting cells at day 0 and every 24 h for 3 days using a the TC10^™^ Automated Cell Counter (Bio-Rad Laboratories). Flow cytometry analysis was performed as previously described [48]. 3T3-L1 and NIH-3T3 cells were seeded in a 6-well culture plate at a density of 8.0 x 10^4^ cells per well. After 2 days post-confluence, cells were incubated in complete medium for the following 16 h in the presence or absence of *CA*de (100 μg/ml) or in DMEM differentiation medium in the presence or absence of *CA*de (100 μg/ml) for 12, 14 and 16 h. The 3T3-L1 cells were harvested and fixed with ethanol 70% a 4 °C overnight. Fixed 3T3-L1 cells were stained with propidium iodide 50 μg/ml (PI; Sigma Aldrich) and incubated with RNase 10 μg/ml (Sigma Aldrich) for 30 min at 37°C in the dark. Fluorescence emitted from cells was measured by flow cytometry (BD FACSAria III, Becton, Dickinson and Company, Franklin Lakes, NJ) using BD FACSDiva software. A total of 10.0 x 10^3^ cells in each sample were analyzed.

### Statistical procedures

For all the analysis, data are expressed as mean ± SD. For experiments with only two groups, comparison was made using two-tailed, unpaired Student’s t-test. For experiments with three or more groups, comparison between groups was determined by one-way analysis of variance (ANOVA) and Bonferroni correction post hoc test was carried out to determine significant differences between specific groups. The GraphPad Software was used to analize data (GraphPad Software, version 6.00 for Windows, La Jolla, CA).

## Results

### *CA*de do not affect 3T3-L1 cell viability at concentrations up to 100 μg/ml

To evaluate the cell viability of the 3T3-L1 cells in response to *CA*de, cells were exposed for 24 h to different *CA*de concentrations (1, 10, 100 and 1000 μg/ml). SRB assays revealed a 10% reduction of viability in cells exposed to the highest *CA*de concentration (1000 μg/ml) compared with un-treated cells; whereas *CA*de did not affect 3T3-L1 cell viability at lower doses (1, 10 and 100 μg/ml; Table 1). The concentration of *CA*de (1000 μg/ml) was therefore excluded for the subsequent experiments.

**Table 1:** Effects of *CA*de on 3T3-L1 pre-adipocytes cell viability.

Cells	*Ca*de (μg/ml)	Cell viability (% over Ctrl)
**3T3-L1 preadipocytes**	/	100.0 ± 0.0
	1000 μg/ml	92.6 ± 2.7***
	100 μg/ml	99.1 ± 3.3
	10 μg/ml	103.9 ± 5.4
	1 μg/ml	103.8 ± 3.7

3T3-L1 pre-adipocytes cultured in 96-well plate were treated with scalar concentrations of *CA*de for 24 h. Citotoxicity was then determined by SRB assay as described under “Materials and Methods”. Data are mean ± SD of determinations from three independent experiments. Statistical analysis was performed using one-way ANOVA.

***p<0.001, *vs*. untreated 3T3-L1 pre-adipocytes.

### *CA*de treatment promotes adipogenesis in 3T3-L1 cells

**Fig 1:**
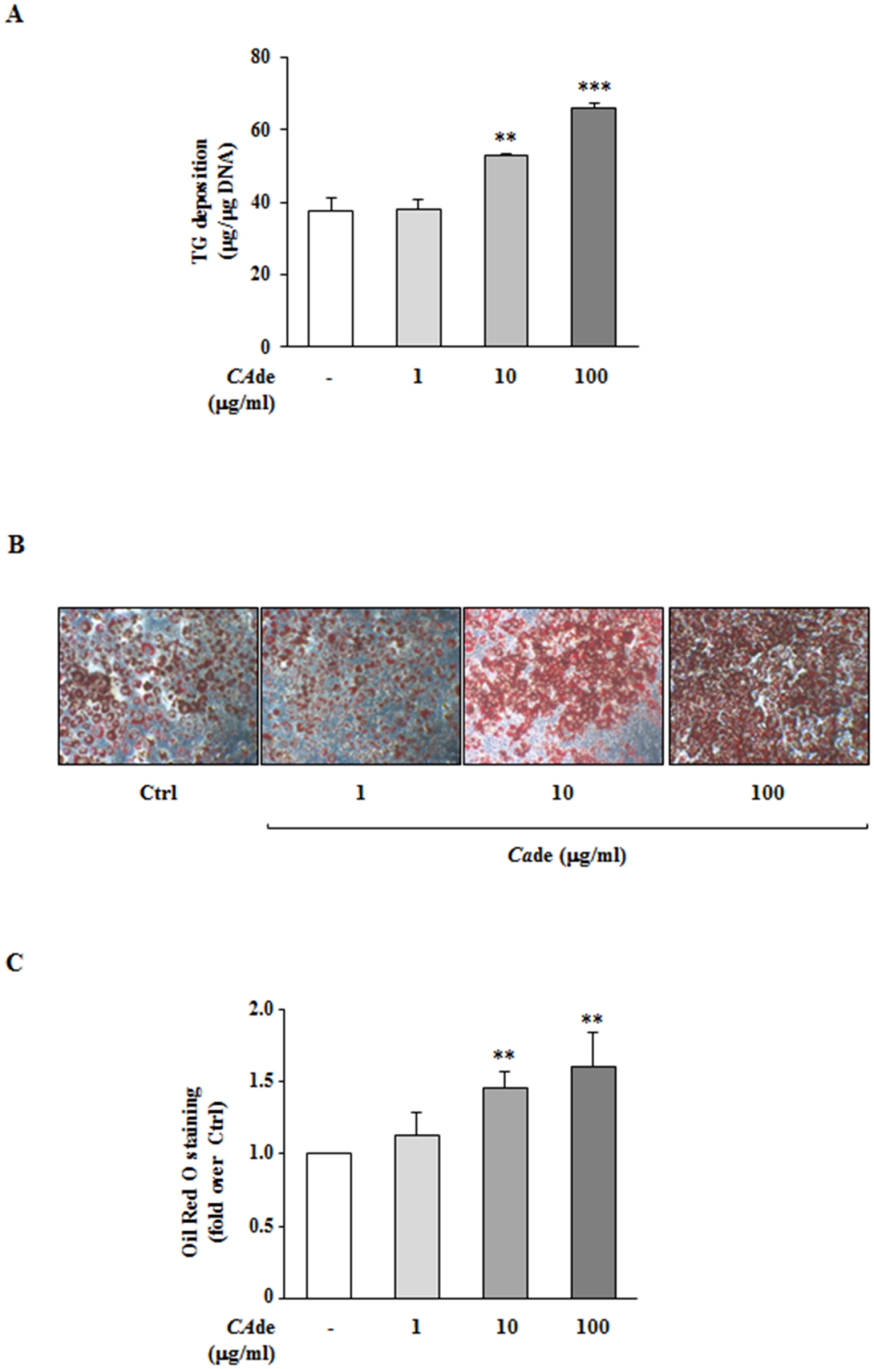
The effects of *CA*de on TG deposition and intra-cellular lipid accumulation in 3T3-L1 adipocytes. 3T3-L1 pre-adipocytes were differentiated into mature adipocytes for 8 days, as described under “Materials and Methods”, in absence (Ctrl) or presence of *CA*de (1, 10 or 100 μg/ml). **A**) Total TG deposition of mature adipocytes. Data are mean ± SD of determinations from three independent experiments. Statistical analysis was performed using one-way ANOVA. **p<0.01, and ***p<0.001, *vs*. Ctrl. Microscopic images (10X magnification) (**B**), and lipid quantization (**C**) of mature adipocytes stained with Oil-Red O. The results are means ± SD of the Oil-Red O absorbance values measured at 490 nm from three independent experiments and are expressed as fold changes over control. Statistical analysis was performed using one-way ANOVA. **p<0.01 *vs*. Ctrl.

To investigate the adipogenic effects of *CA*de, 3T3-L1 pre-adipocytes were differentiated into mature adipocytes with an adipogenic cocktail in presence or absence of various doses of *CA*de (1, 10 and 100 μg/ml) for 8 days, and TG deposition and lipid accumulation of cells were measured, at the end of the treatment, as events associated with terminal adipocyte differentiation. *CA*de treatment at the doses of 10 and 100 μg/ml, but not at the lower concentration of 1 μg/ml, enhanced of about 40 and 70%, respectively, the TG deposition of treated 3T3-L1 adipocytes compared with the TG deposition of control adipocytes (Fig 1A). Concurrently, as shown by Oil-Red O staining, in presence of *CA*de at a dose of 10 or 100 μg/ml for 8 days, but not at 1 μg/ml, cells displayed a dose dependent increase of the intra-cellular lipid accumulation compared to control adipocytes (Fig 1B and 1C). DNA content of 3T3-L1 cells treated with all the three concentrations of *CA*de (1, 10 and 100 μg/ml) was unchanged compared with the untreated control cells (https://figshare.com/s/a67a96577408da7e3a43). Altogether, these data suggest that the treatment with *CA*de promotes 3T3-L1 adipogenesis, in terms of TG and lipid accumulation. From now on the dose of 100 μg/ml *CA*de, which showed the more adipogenic effect on 3T3-L1 cell, was used for the all subsequent experiments.

### *CA*de up-regulates the expression of master regulators of adipogenesis in 3T3-L1 cells

**Fig 2:**
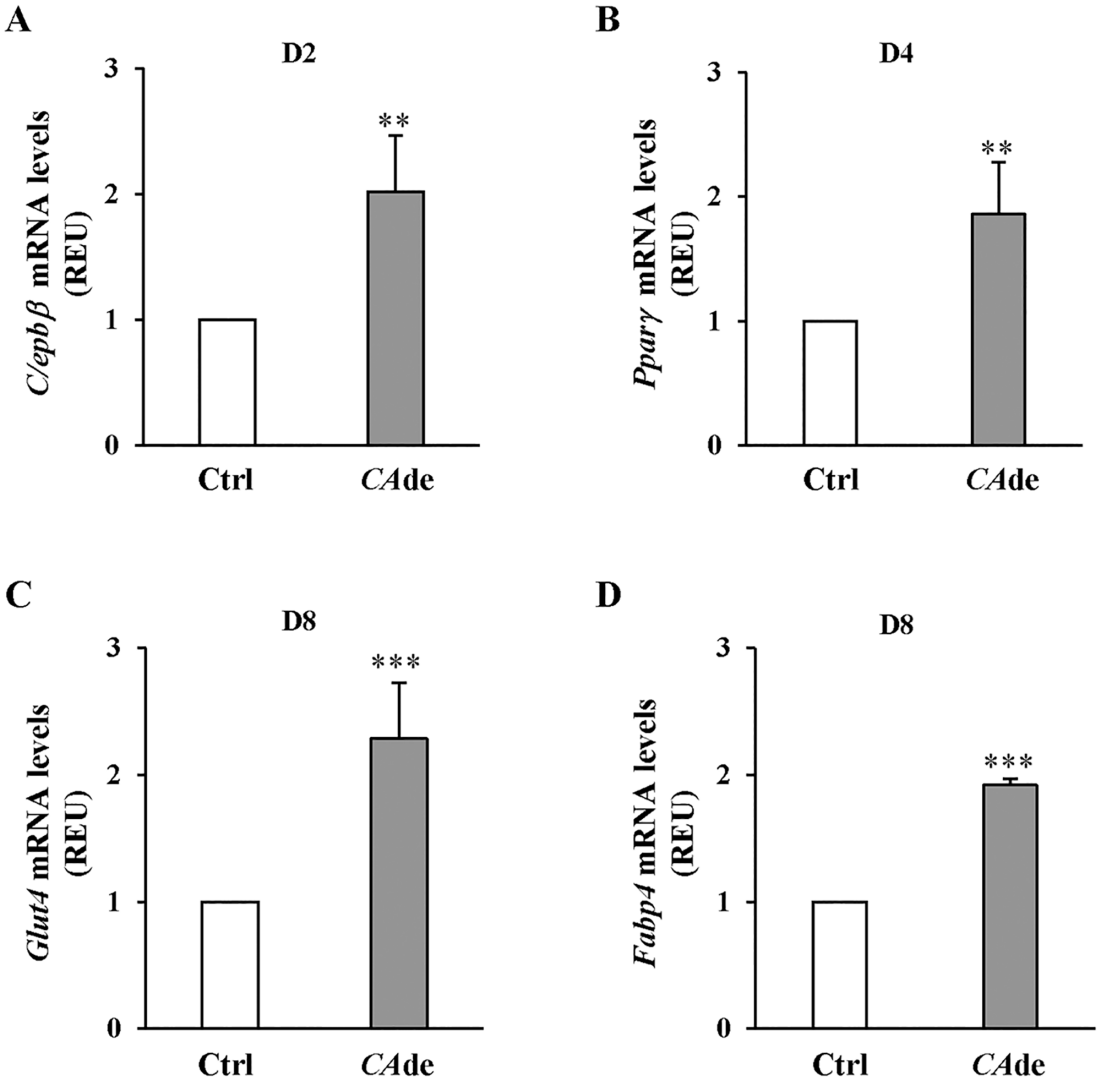
The effects of *CA*de on gene expression during adipogenesis in 3T3-L1 cells. 3T3-L1 pre-adipocytes were differentiated into mature adipocytes for 8 days, as described under “Materials and Methods”, in absence (Ctrl) or presence of *CA*de (100 μg/ml). At the indicated time points, cells were collected for the extraction of RNA. qPCR was performed to detect the mRNA expression of (**A**) *C/ebpβ* at D2, (**B**) *Pparγ* at D4, and (**C**) *Glut4* and (**D**) *Fapb4* at D8 of the adipogenesis. Results are means ± SD of three independent experiments and are expressed as relative changes over control. Statistical analysis was performed using Student’s t-test. **p<0.01, and ***p<0.001 *vs*. Ctrl at D2, D4 or D8.

To establish how *CA*de enhances adipogenesis in 3T3-L1 cells, we measured the expression of adipogenic markers at day 2, 4 and 8 of the differentiation process. The mRNA levels of the *CCAAT/enhancer binding protein beta* (*C/ebpβ)* at day 2 were increased by 2-fold (Fig 2A; Figure A in S1 File), the expression levels of the *Peroxisome proliferator activated receptor gamma* (*Pparγ*) at day 4 were increased by 1.8-fold (Fig 2B; Figure A in S1 File), and of the *solute carrier family 2 (facilitated glucose transporter) member 4* (*Glut4*) and the *Fatty acid binding protein* 4 (*Fabp4*) at day 8 were increased by 2.3- and 1.9-fold, respectively, (Fig 2C and 2D; Figure A in S1 File) in 3T3-L1 cells differentiated in presence of *CA*de compared with control cells. Differently, no changes in the mRNA expression levels of *C/ebpδ* at day 2 and *C/ebpα* at day 4 were found among cells differentiated in presence or absence of *CA*de (https://figshare.com/s/a67a96577408da7e3a43). These findings suggest that *CA*de enhances the expression of specific master regulator genes of adipogenesis as early as 2 days upon induction of differentiation.

### *CA*de promotes the early stages of adipocyte differentiation and enhances insulin-mediated glucose uptake in 3T3-L1 cells

**Fig 3:**
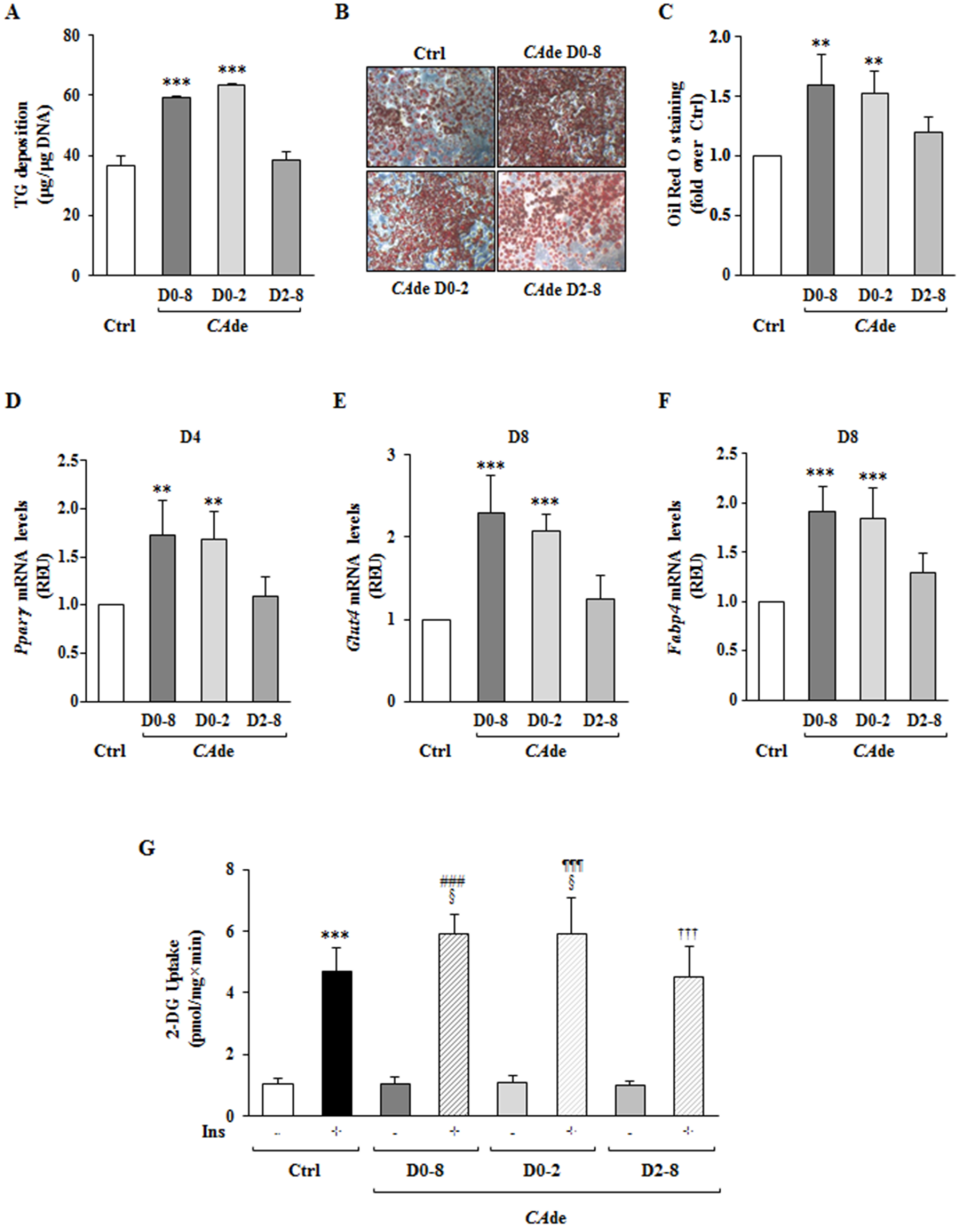
The effects of *CA*de on the early stage of adipogenesis in 3T3-L1 cells. 3T3-L1 pre-adipocytes were differentiated into mature adipocytes for 8 days, as described under “Materials and Methods”, in absence (Ctrl) or presence of *CA*de (100 μg/ml) from D0 to D2 (D0-D2), from D2 to D8 (D2-D8) and from D0 to D8 (D0-D8). **A**) Total TG deposition of mature adipocytes. Data are mean ± SD of determinations from three independent experiments. Statistical analysis was performed using one-way ANOVA. ***p<0.001, *vs*. Ctrl. Microscopic images (10X magnification) (**B**), and lipid quantization (**C**) of mature adipocytes stained with Oil-Red O. The results are means ± SD of the Oil-red O absorbance values measured at 490 nm from three independent experiments and are expressed as fold changes over control. Statistical analysis was performed using one-way ANOVA. **p<0.01 *vs*. Ctrl. qPCR was performed to detect the mRNA expression of (**D**) *Pparγ* at D4, and (**E**) *Glut4* and (**F**) *Fapb4* at D8 of the adipogenesis. Results are means ± SD of three independent experiments and are expressed as relative changes over control. Statistical analysis was performed using one-way ANOVA. *p<0.05, **p<0.01, and ***p<0.001 *vs*. Ctrl at D2, D4 or D8. **G**) The uptake of 2-DG was then evaluated in mature adipocytes upon stimulation with insulin (100 nmol/l; Ins) for 30 min. The results are means ± SD of three independent experiments. Statistical analysis was performed using one-way ANOVA. ***p<0.001 *vs*. Ctrl—Ins; ^###^*p*<0.001, *CA*de D0-D8 + Ins *vs*. *CA*de D0-D8—Ins; ^*¶¶¶*^*p*<0.001, *CA*de D0-D2 + Ins *vs*. *CA*de D0-D2—Ins; ^†††^*p*<0.001, *CA*de D2-D8 + Ins *vs*. *CA*de D2-D8—Ins; ^§^*p*<0.05 *vs*. Ctrl + Ins.

The pre-adipocytes differentiation into mature adipocytes includes cell commitment and mitotic clonal expansion (MCE), occurring within the first 48 h upon adipogenic induction, and intermediate and late cell differentiation phases, proceeding from D2 to D8 [32]. To investigate whether *CA*de modulates specific phases of adipose cell differentiation, 3T3-L1 cells were differentiated in the presence of *CA*de from day 0 to day 2 (D0-D2) or from day 2 to day 8 (D2-D8) of adipocyte differentiation. At the end of the differentiation process, similarly to adipocytes matured in presence of *CA*de for 8 days (D0-D8), cells exposed early to *CA*de (D0-D2) showed a significant increase of both TG deposition and intra-cellular lipid accumulation compared with control mature adipocytes (Fig 3A, 3B and 3C). Also, gene expression of *Pparγ* at D4, and *Glut4* and *Fabp4* at D8 was augmented in these conditions (Fig 3D, 3E and 3F; Figure A in S1 File). On the contrary, in cells treated with *CA*de from D2 to D8 (D2-D8) no increases of TG levels and lipid droplet formation at D8 (Fig 3A, 3B and 3C) and of the mRNA expression of *Pparγ*, *Glut4* and *Fabp4* of adipocyte differentiation (Fig 3D, 3E and 3F; Figure A in S1 File), were observed compared with control adipocytes. These data indicate that the effectiveness of *CA*de treatment is restricted within the first 48 h of the differentiation process in 3T3-L1 cells.

Then, to figure out whether the treatment with *CA*de may also improve adipocyte functionality, we investigated the basal and the insulin-mediated 2-DG uptake in mature 3T3-L1 adipocytes differentiated in presence or absence of *CA*de from D0-D8, from D0-D2 or from D2-D8. As expected, in adipocytes differentiated without *CA*de, 100 nmol/l insulin for 30 min induced by 4.5-fold the 2-DG uptake compared with the basal 2-DG uptake (Fig 3G). On the other hand, cells differentiated in presence of *CA*de from D0-D8 showed a 5.7-fold increase of the insulin-mediated 2-DG uptake compared with the basal 2-DG uptake (Fig 3G). Interestingly, the cells treated with *CA*de from D0 to D2, similarly to the cells exposed to *CA*de from D0 to D8, also showed an increase of the insulin-mediated 2-DG uptake compared with control cells (Fig 3G). On the contrary, the insulin-mediated 2-DG uptake of cells treated with *CA*de from day 2 to day 8 was similar to the glucose uptake of unexposed control cells (Fig 3G). These data suggest that the treatment with *CA*de, even restricted within the first 48 h of the differentiation process, also promotes glucose uptake in response to insulin stimulation.

### *CA*de modulates cell cycle progression during MCE in 3T3-L1 cells

**Fig 4:**
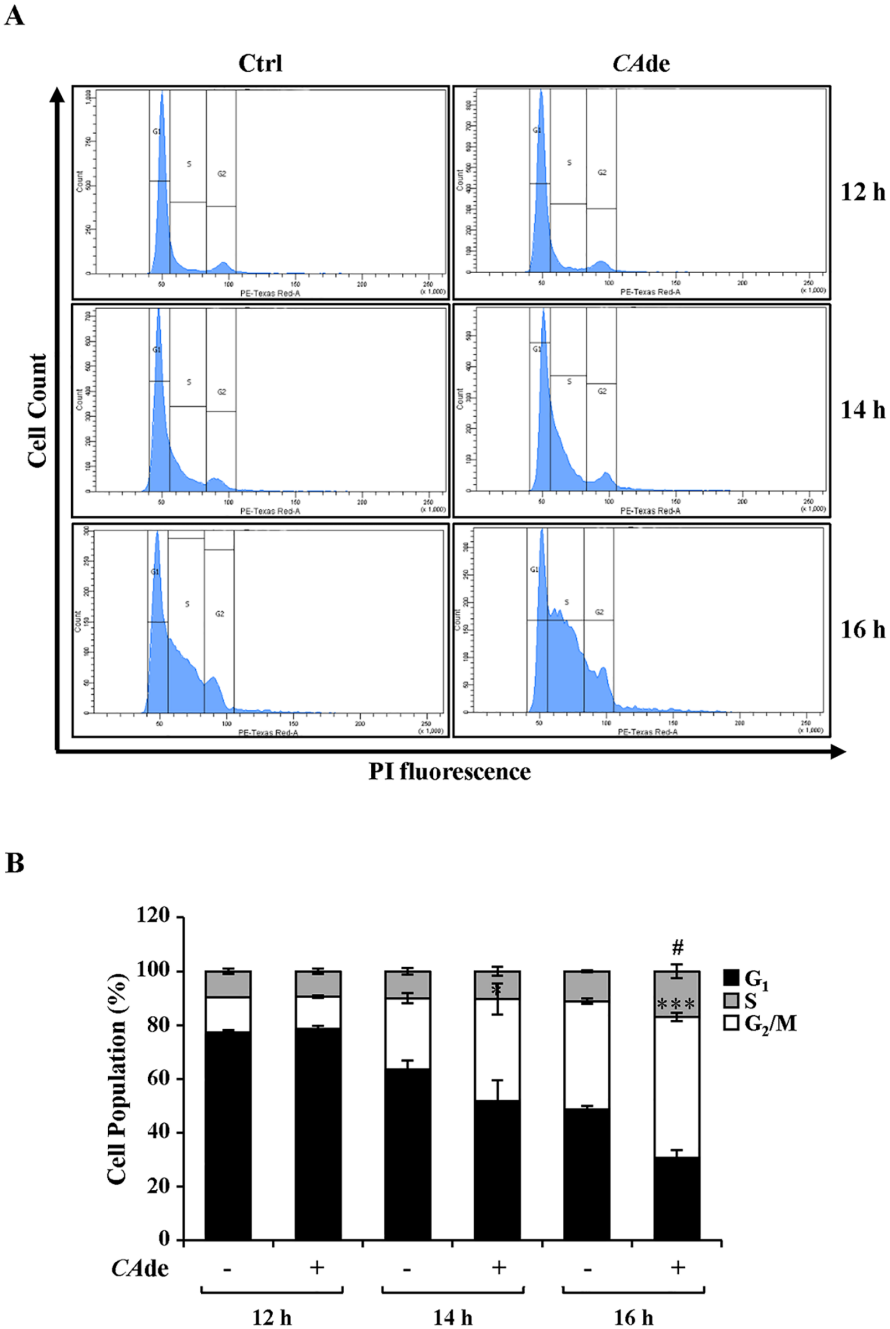
The effects of *CADE* on cell cycle progression during adipogenesis in 3T3-L1. MCE was induced in 3T3-L1 pre-adipocytes with differentiation medium, as described under “Materials and Methods”. Cells were then harvested at 12, 14, and 16 h after the initiation of differentiation in absence (Ctrl) or presence of *CA*de (100 μg/ml) and stained with PI solution for flow cytometer cell cycle analysis. **A**) Histograms of cell cycle distribution in G_0_/G_1_, S or G_2_/M phases. **B**) Quantitative analysis of cell cycle distribution. The results are means ± SD of three independent experiments. Statistical analysis was performed using Student’s t-test *p<0.05, and ***p<0.001, *CA*de S Phase *vs*. Ctrl S Phase; ^#^*p*<0.05, *CA*de G_2_/M Phase *vs*. Ctrl G_2_/M Phase.

After exposure to the adipogenic cocktail, post-confluent growth-arrested pre-adipocytes undergo two successive mitoses over 2 days, which is indicated as MCE [33]. Therefore, to evaluate whether *CA*de promotes adipocyte differentiation in 3T3-L1 cells by inducing changes in cell cycle progression during MCE, cell cycle distribution was analyzed by flow cytometry. At 12 h post induction no differences of the cell distribution in the G_0_/G_1_, S and G_2_/M phases were observed among 3T3-L1 pre-adipocytes exposed or not to *CA*de (Fig 4A and 4B). At 14 h post induction *CA*de treatment decreased the number of cells in the G_0_/G_1_ phase and concomitantly increased the number of cells in the S phase (S phase: Ctrl, 26.5% ± 5.7% vs *CA*de, 38.0% ± 1.0%; *p*<0.05; Fig 4A and 4B). At 16 h post induction, *CA*de not only decreased the number of cells in the G0/G1 phase but also increased the number of cells in the S and G_2_/M phases (S phase: Ctrl, 40.2% ± 1.5% vs *CA*de, 52.2 ± 0.9%; *p*<0.001; G_2_/M phase: Ctrl, 11.1% ± 2.5% vs *CA*de, 17.0 ± 2.2%; *p*<0.05; Fig 4A and 4B). Interestingly, *CA*de did not induce enhancement of MCE in the uncommitted mouse embryonic fibroblasts NIH-3T3 cells (Table B in S1 File). Furthermore, the cell cycle distribution of both post-confluent growth-arrested 3T3-L1 and NIH-3T3 cells just treated with *CA*de for 16 h in the absence of the adipogenic cocktail did not differ compared with the untreated control cells (Table B in S1 File). In addition to this, *CA*de treatment did not change both 3T3-L1 and NIH-3T3 cell proliferation, as well (Table B in S1 File). These results thus suggest that *CA*de exhibits pro-adipogenic properties specifically in 3T3-L1 cells by modulating and promoting their cell cycle progression in the early stage of the differentiation process.

### *CA*de early enhances *C/ebpβ* expression through the activation of the cAMP response element-binding protein (CREB) in 3T3-L1 cells

**Fig 5:**
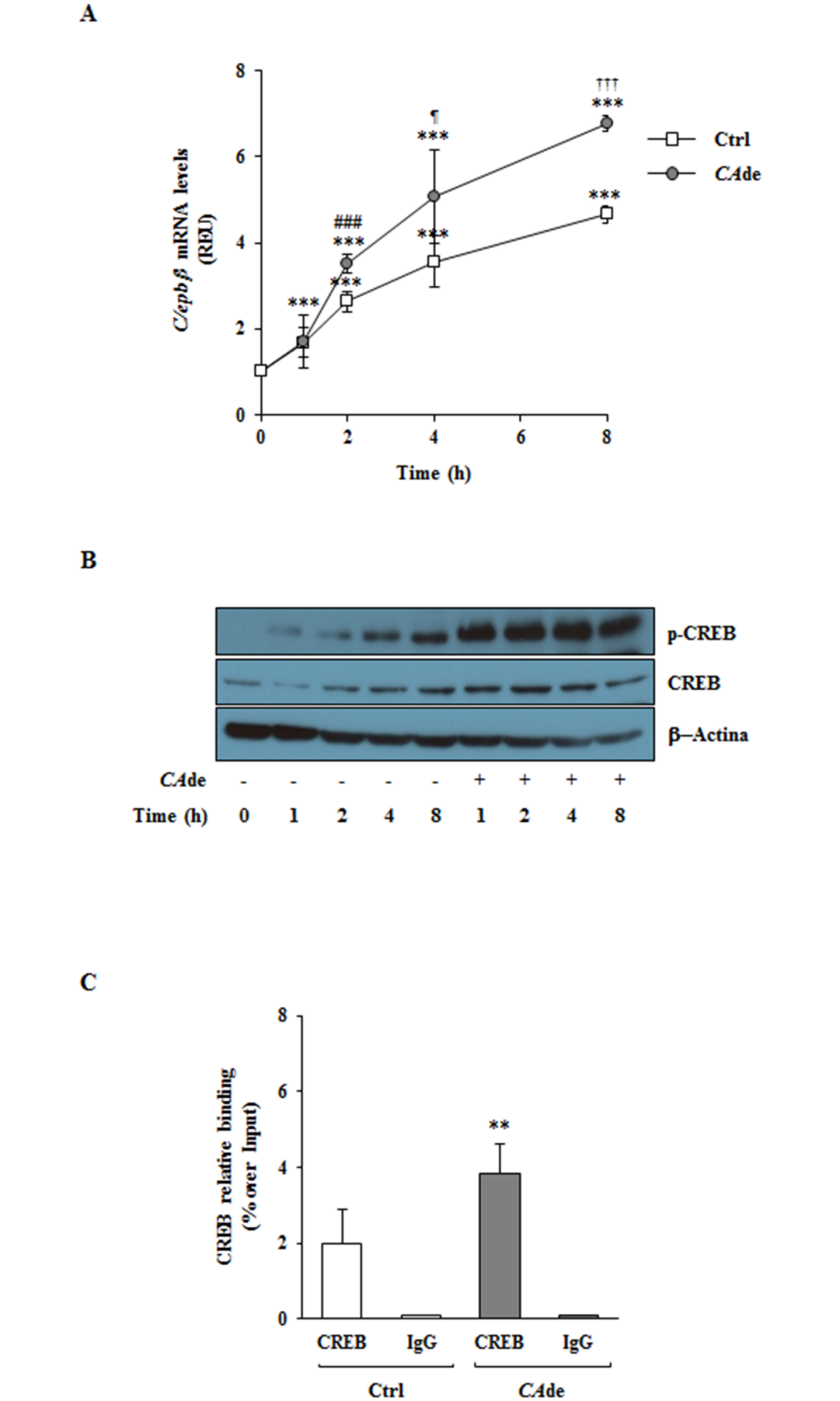
The effects of *CADE* on *C/ebpβ* gene expression and CREB activation during the early stage of adipogenesis in 3T3-L1. Adipogenesis was induced in 3T3-L1 pre-adipocytes with the differentiation medium (MDI), as described under “Materials and Methods”. Cells were then harvested at 1, 2, 4 and 8 h after the initiation of differentiation in absence (Ctrl) or presence of *CA*de (100 μg/ml) and processed for qPCR and western blot analysis. **A**) qPCR of *C/ebpβ* mRNA expression. Results are means ± SD of three independent experiments and are expressed as relative changes over control. Statistical analysis was performed using one-way ANOVA. ***p<0.01, *vs*. 3T3-L1 cells at 0 h; ^###^*p*<0.001, *CA*de 2 h *vs*. Ctrl 2 h; ^*¶*^*p*<0.05, *CA*de 4 h *vs*. Ctrl 4 h; ^†††^*p*<0.001, *CA*de 8 h *vs*. Ctrl 8 h. **B**) The representative western blot show levels of the total and Ser^133^ phosphorylated form of the cAMP response element-binding protein (CREB) and of the β-Actin protein. **C)** CREB protein binding on *C/ebpβ* promoter was evaluated by ChIP analysis on 3T3-L1 cells harvested at 4 h after the initiation of differentiation in absence (Ctrl) or presence of *CA*de (100 μg/ml). ChIP enrichment is relative to input chromatin. Data are expressed as mean ± SD of values from at least three independent experiments. Statistical analysis was performed using Student’s t-test. **p<0.01, *CA*de 4 h *vs*. Ctrl 4 h.

C/ebpβ plays a basic role in the regulation of the adipogenesis and is necessary for the initiation of the MCE in 3T3-L1 cells [49–54]. Therefore, to investigate whether and how *CA*de directly modulates *C/ebpβ*, we measured both the *C/ebpβ* gene expression and the activation state of its transcriptional regulator CREB [55] in the early times post the adipogenic induction of 3T3-L1 cells with the differentiation medium (MDI). As expected, in the control cells, MDI induced already upon 1 h a 1.7-fold increase of the mRNA expression of *C/ebpβ*, which levels furtherly raised at 2 and 4 h and reached its maximal expression at 8 h post treatment (Fig 5A). Concurrently, in these cells MDI also led to CREB activation, measured as phosphorylation of the Ser^133^, which was evident as early as 1 h and persisted up to 8 h following the adipogenic induction (Fig 5B). Interestingly, *CA*de treatment additionally enhances the effect of MDI on *C/ebpβ* expression and CREB activation (Fig 5A and 5B). Indeed, in *CA*de-treated cells the *C/ebpβ* gene expression resulted to be increased of about 30, 40 and 45% at 2, 4 and 8 h upon MDI and *CA*de induction, respectively, compared with its expression at the same time points in the MDI treated control cells (Fig 5A). These data were also paralleled by a strong induction of the CREB activation, which phosphorylation levels were higher as early as 1 h after the combined MDI and *CA*de treatments and still remained more elevevated up to 8 h following the treatments compared with the phosphorylation levels of the MDI treated control cells (Fig 5B). Furthermore, the specific recruitment of CREB to its binding sites (Site 1, -111/-101 bp from the *C/ebpβ* gene transcription starting site, TSS, and Site 2, -65/-55 bp from the TSS) on the *C/ebpβ* promoter investigated by ChIP analysis showed a 2-fold increase in CREB binding to the *C/ebpβ* promoter at 4 h in 3T3-L1 cells differentiated in the presence of *CA*de compared with the MDI treated cells (Fig 5C). These data indicate that *CA*de enhances the effect of the adipogenic induction in 3T3-L1 cells at least in part by early promoting and sustaining the *C/ebpβ* expression through the increased activation of the transcription factor CREB.

### Effects of single compounds on *C/ebpβ* gene expression in 3T3-L1 cells

**Fig 6:**
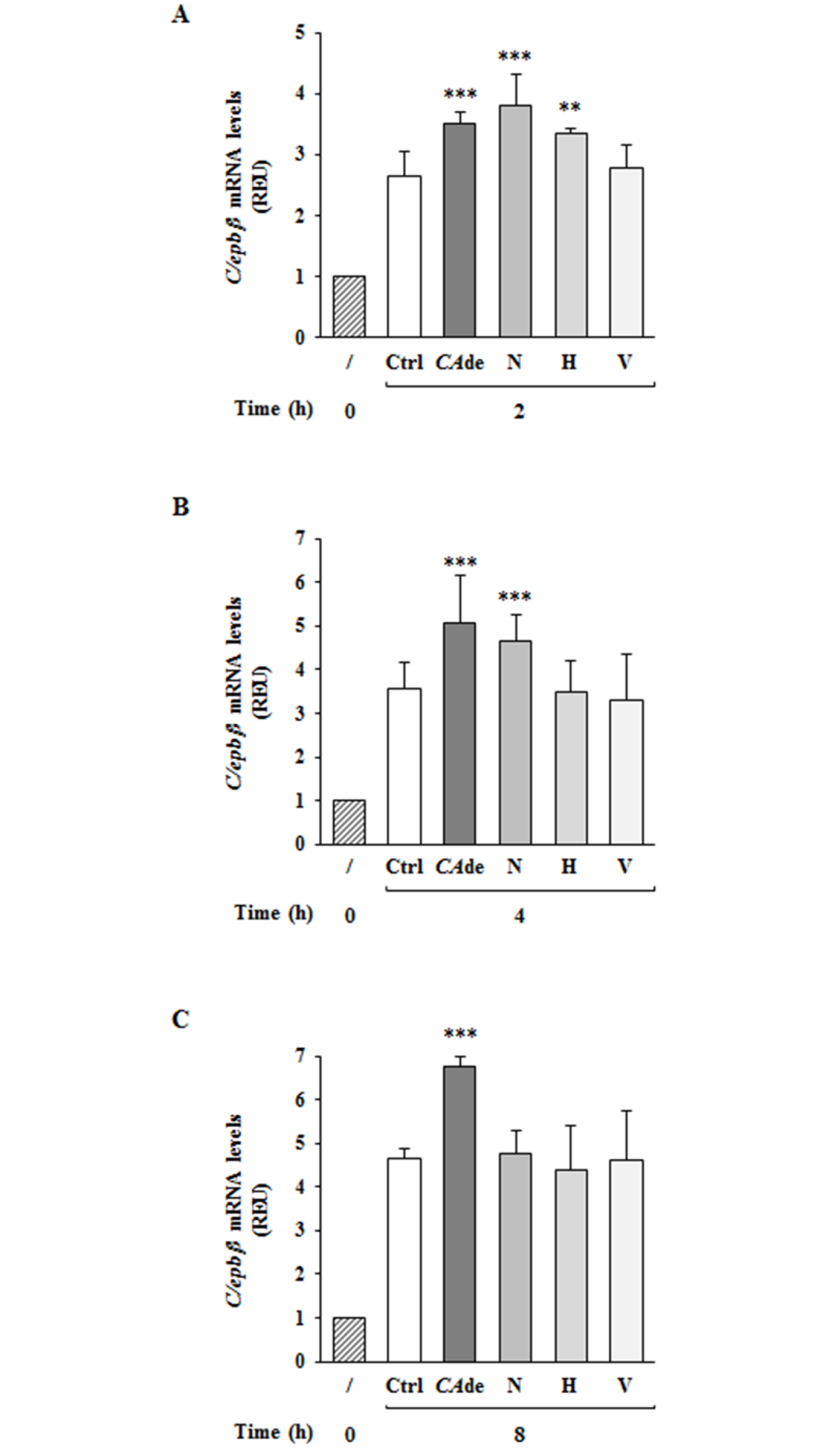
The effects of single flavonoids on *C/ebpβ* gene expression during the early stage of adipogenesis in 3T3-L1. Adipogenesis was induced in 3T3-L1 pre-adipocytes with the differentiation medium (MDI). Cells were then harvested at 2, 4 and 8 h after the initiation of differentiation in the absence (Ctrl) or presence of 6.7 μg/ml narirutin (N) or 3.9 μg/ml hesperidin (H) or 5.5 μg/ml vicenin-2 (V). Cells treated with *CA*de (100 μg/ml) were also used. At the indicated time points, cells were collected for the extraction of RNA. qPCR was performed to detect the mRNA expression of *C/ebpβ* at 2h (**A**), 4h (**B**), and 8h (**C**) upon adipogenesis. Results are means ± SD of three independent experiments and are expressed as relative changes over control cells at time 0. Statistical analysis was performed using one-way ANOVA. **p<0.01, and ***p<0.001 *vs*. Ctrl at 2, 4 or 8 h.

Finally, to investigate the hypothesis that the effects of *CA*de on *C/ebpβ* gene expression may be dependent to the activity of specific molecules within *CA*de, a qualitative and quantitative analysis of compounds in *CA*de was performed. From this analysis resulted that among 17 identified metabolites, the O-glycoside flavanones narirutin and hesperidin, which concentration for gram of *CA*de is 67.51 and 39.05 mg, respectively, and the C-glycoside flavone vicenin-2 (55.56 mg/g of *CA*de) were the most abundant (Table A in S1 File). The effects of these three flavonoids on *C/ebpβ* gene expression in the early times post the adipogenic induction of 3T3-L1 cells with MDI was thus investigated. Interestingly, similarly to cells treated with *CA*de, cells exposed to narirutin showed a stronger increase of the mRNA expression of *C/ebpβ* at 2 h and 4 h post adipogenic induction compared to control cells (Fig 6A and 6B), but at 8 h the *C/ebpβ* levels were comparable among control and narirutin treated cells (Fig 6C). Differently from narirutin, hesperidin significantly enhanced *C/ebpβ* expression of 3T3-L1 cells only at 2 h post adipogenic induction, but not at 4 and 8 h, compared to control cells (Fig 6A, 6B and 6C), while vicenin-2 did not affect *C/ebpβ* gene expression levels of 3T3-L1 cells neither at 2, 4 or 8 h upon MDI (Fig 6A, 6B and 6C).

## Discussion

T2D is a growing problem for both developed and developing countries and represents a burden on healthcare systems as well as individuals [1, 2]. It is primarily the results of excess body fat and physical inactivity, and is caused by a combination of abnormalities affecting the metabolic and/or endocrine function of both pancreatic beta cells and target tissues, including WAT [2–4]. WAT, consisting of several cell types including mature adipocytes, is one of the largest organs in humans which mainly works as reserve of lipids to store and mobilize according to the energy needs, and as endocrine organ contributing to the complex homeostatic regulation of the energy intake, and of the glucose and lipid metabolisms [36, 37]. Several evidence sustains that its dysfunction contributed to the development of metabolic disorders, such as T2D, as well as, other pathologies, like atherosclerosis and cardiovascular disease [3, 4, 56]. *E*.*g*., adipocyte hypertrophy, which is characterized by restricted adipogenesis and increased fat cell size, is an adaptive process, usually occurring as a response to long-term positive energy balance, that is accompanied by disturbances in lipid metabolism, alterations in adipokine secretion, and low grade inflammation [5–7]. The lasts have detrimental effects on insulin sensitivity, promoting insulin resistance both locally in the adipose tissue and whole-body by leading to ectopic fat accumulation in non-adipose depots, such as liver, skeletal muscle and vessels [5–7]. Therefore, the generation of pharmaceutical and/or nutraceutical compounds targeted to improve WAT functional capacity represents an alternative and complementary strategy for the prevention and/or treatment of T2D.

In this context, we have intensely studied the potential nutraceutical effects of *CA*de, a preparation of dry extracts obtained from *Citrus aurantium* L. fruit juice *in vitro*, on the regulation of 3T3-L1 cells adipocyte differentiation and function. Adipogenesis is a highly controlled cell differentiation process. It consists of several stages, including cell commitment and MCE, which occur early from D0 to D2 of the adipogenic process, and intermediate and late cell differentiation phases, proceeding from D2 to D8 [32, 57]. Adipogenesis, furthermore, requires the sequential activation of a series of transcription factors, such as the *C/ebp* gene family (*C/ebp-α*, *-β* and *-δ*) and *Pparγ* [58, 59]. The last one is the master regulator of the adipocyte differentiation and modulates the expression of several genes during the whole process [60]. Transcriptional activation of *Pparγ* is required for the achievement of a complete fat cell maturity through the transactivation of specific genes such as the *insulin receptor* and the insulin-dependent glucose transporter *Glut4*, which are required to transduce the insulin signaling and to promote the glucose uptake [32], as well as *Fabp4*, which encodes a protein able to bind long-chain fatty acids and other hydrophobic ligands facilitating the transport and metabolism of fatty acids inside the adipocytes [61]. Our study revealed that the treatment of 3T3-L1 pre-adipocytes with *CA*de enhances adipogenesis *in vitro*, as shown by the increased TG and lipid accumulation and by the increased gene expression of the adipocyte-specific markers, *C/ebpβ*, *Pparγ*, *Glut4* and *Fabp4*. Furthermore, we also disclosed that *CA*de treatment is able to robustly improve the 2-DG uptake upon insulin stimulation. Differently from our findings, Kim *et al*. have previously reported that a preparation of flavonoids extracted from *Citrus aurantium* L. fruit peel suppressed adipogenesis in 3T3-L1 cells by down-regulating *C/ebpβ* expression and subsequently inhibiting the activation of *Pparγ* and *C/ebpα* [61, 62, 63]. However, the observed discrepancy among their and our findings is not surprising and may be proprably ascribed to diversities in the nutritional products among the *Citrus aurantium* L. fruit peel and fruit juice extract preparations. Jabri Karoui *et al*. have indeed reported qualitative and quantitative differences in the polyphenolyc composition among preparations obtained from *Citrus aurantium* L. fruit peel or fruit juice [38]. In accordance to this, a comparison among *CA*de and the *Citrus aurantium* Flavonoids (CAF) extract used by Kim GS and collegues [61, 63] revealed substantial differences in the flavonoid composition among the two preparations. *CA*de is indeed enriched in the O-glucoside flavonones, hesperidin and narirutin, and the C-glucoside flavane vicenin-2. In particular, the first one is about 200-times more concentred in *CA*de compared with CAF, while the other two are present only on *CA*de preparation. However, when the effect of each one of these three flavonoids on *C/ebpβ* gene regulation was investigated, none of them reached effects comparable to *CA*de. Indeed, narirutin and hesperidin upregulate *C/ebpβ* mRNA levels only within the first 4 hours upon adipogenic induction, whilst vicenin-2 has no effects at all. We also looked at the presence of the sympathomimetic agent p-synephrine (or its metabolite p-octopamine) within *CA*de. Its content was not detectable, suggesting its absence or presence at very low concentrations in *CA*de. This may be related to the ripening of the fruits employed for *CA*de preparation. Indeed, p-synephrine is very abundant in extracts from *Citrus aurantium* L. unripe fruits [64–65]. These findings thus support the concept that the beneficial effects of *CA*de on adipocyte differentiation capacity are attributable to the specific and unique distribution of bioactive compounds within *CA*de.

Here, we have furthermore demonstrated that *CA*de improves adipocyte differentiation and function of the 3T3-L1 cells by acting on the early stages of adipogenesis. Indeed, when adipogenesis was investigated culturing pre-adipocytes with *CA*de at the temporal windows, D0-D2 and D2-D8, only the treatment with dry extracts for the first 48 h was sufficient to increase the expression of *Pparγ*, *Glut4* and *Fabp4*, to sustain the terminal adipocyte differentiation and to nurture the glucose uptake to levels comparable to that observed into cells exposed to a continuous treatment with *CA*de. Several evidence nowadays indicates that the transcription factor C/ebpβ plays a pivotal role in the regulation of the adipogenesis and its activation is a prerequisite for the initiation of the MCE in the adipocyte-differentiation program [49–52]. Tang *et al*. have recently demonstrated that mouse embryo fibroblasts (MEFs) from *C/ebpβ*^(-/-)^ mice neither undergo MCE nor differentiate into mature adipocytes, while the forced expression of C/ebpβ in the same MEFs restores both MCE and adipogenesis [52]. C/ebpβ is expressed as early as 2–4 h after induction, but it acquired its DNA binding activity and ability to activate gene expression at 14–16 h upon adipogenic induction in concomitance with the beginning of MCE [53, 54]. Accordingly with the latter observations, here we also demonstrated in 3T3-L1 pre-adipocytes that *CA*de strongly up-regulates, simultaneously with the CREB activation and binding to the *C/ebpβ* gene promoter, the *C/ebpβ* expression at 2, 4 and 8 h post the adipogenic induction and drives cell cycle progression during MCE at 14–16 h post MDI treatment. Therefore, based on these findings the effects of *CA*de during early adipogenesis is, at least in part, due or associated with the specific activation of *C/ebpβ*.

## Conclusions

Our work reveals the nutraceutical properties of our preparation of *Citrus aurantium* L. fruit juice dry extracts (*CA*de) on the fat cell functional capacity, in terms of enhanced adipocyte differentiation and function *in vitro*. These data furthermore provide solid basis for the elaboration of nutraceutical strategies aimed at the development of products derived from *CA*de, which may improve the functional capacity of the WAT and may thus be effective for the prevention and/or treatment of T2D.

## Supporting information

S1 File Supporting information file. It includes Materials and Methods A, Table A, Table B, and Figure A in S1 File. **Materials and Methods A in S1 File. UHPLC-PDA and FT-ICR-MS conditions, and identification and quantification of flavonoids. Table A in S1 File. Qualitative and quantitative flavonoid profile of *CA*de. Table B in S1 File. Effects of *CA*de on 3T3-L1 and NIH-3T3 cell cycle progression and cell proliferation. Figure A in S1 File. The effects of *CA*de on gene expression during adipogenesis in 3T3-L1 cells**.
(PDF)

## References

[1] Diabetes Fact sheet. Reviewed November 2016. World Health Organization. http://www.who.int/mediacentre/factsheets/fs312/en/.

[2] Alberti KG, Zimmet PZ. Definition, diagnosis and classification of diabetes mellitus and its complications. Part 1: diagnosis and classification of diabetes mellitus provisional report of a WHO consultation. Diabet Med. 1998; 15(7): 539–553. doi: 10.1002/(SICI)1096-9136(199807)15:7<539::AID-DIA668>3.0.CO;2-S 9686693 10.1002/(SICI)1096-9136(199807)15:7<539::AID-DIA668>3.0.CO;2-S

[3] Pellegrinelli V, Carobbio S, Vidal-Puig A. Adipose tissue plasticity: how fat depots respond differently to pathophysiological cues. Diabetologia. 2016;59(6): 1075–1088. doi: 10.1007/s00125-016-3933-4 27039901 10.1007/s00125-016-3933-4 PMC4861754

[4] Fox KA, Després JP, Richard AJ, Brette S, Deanfield JE; IDEA Steering Committee and National Co-ordinators. Does abdominal obesity have a similar impact on cardiovascular disease and diabetes? A study of 91,246 ambulant patients in 27 European countries. Eur Heart J. 2009;30(24): 3055–3063. doi: 10.1093/eurheartj/ehp371 19778928 10.1093/eurheartj/ehp371

[5] Guilherme A, Virbasius JV, Puri V, Czech MP. Adipocyte dysfunctions linking obesity to insulin resistance and type 2 diabetes. Nat Rev Mol Cell Biol. 2008;9(5): 367–377. doi: 10.1038/nrm2391 18401346 10.1038/nrm2391 PMC2886982

[6] Lönn M, Mehlig K, Bengtsson C, Lissner L. Adipocyte size predicts incidence of type 2 diabetes in women. FASEB J. 2010;24(1): 326–331. doi: 10.1096/fj.09-133058 19741173 10.1096/fj.09-133058

[7] Gustafson B, Hammarstedt A, Hedjazifar S, Smith U. Restricted adipogenesis in hypertrophic obesity: the role of WISP2, WNT, and BMP4. Diabetes. 2013;62(9): 2997–3004. doi: 10.2337/db13-0473 23970518 10.2337/db13-0473 PMC3749334

[8] Anderson JW, Pasupuleti VK (Eds). Nutraceuticals and Diabetes Prevention and Management, in Nutraceuticals, Glycemic Health and Type 2 Diabetes. Wiley-Blackwell, Oxford, UK; 2008.

[9] Nasri H, Baradaran A, Shirzad H, Rafieian-Kopaei M. New concepts in nutraceuticals as alternative for pharmaceuticals. Int J Prev Med. 2014;5(12): 1487–1499. 25709784 PMC4336979

[10] Ramaa CS, Shirode AR, Mundada AS, Kadam VJ. Nutraceuticals-an emerging era in the treatment and prevention of cardiovascular diseases. Curr Pharm Biotechnol. 2006;7(1): 15–23. 16472130 10.2174/138920106775789647

[11] Nair HB, Sung B, Yadav VR, Kannappan R, Chaturvedi MM, Aggarwal BB. Delivery of antiinflammatory nutraceuticals by nanoparticles for the prevention and treatment of cancer. Biochem Pharmacol. 2010;80(12): 1833–1843. doi: 10.1016/j.bcp.2010.07.021 20654584 10.1016/j.bcp.2010.07.021 PMC2974020

[12] Mecocci P, Tinarelli C, Schulz RJ, Polidori MC. Nutraceuticals in cognitive impairment and Alzheimer”s disease. Front Pharmacol. 2014;5: 147 doi: 10.3389/fphar.2014.00147 25002849 10.3389/fphar.2014.00147 PMC4066843

[13] Dey L, Attele AS, Yuan CS. Alternative therapies for type 2 diabetes. Altern Med Rev. 2002;7: 45–58. 11896745

[14] Grover JK, Yadav S, Vats V. Medicinal plants of India with anti-diabetic potential. J Ethnopharmacol. 2002;81: 81–100. 12020931 10.1016/s0378-8741(02)00059-4

[15] Leung L, Birtwhistle R, Kotecha J, Hannah S, Cuthbertson S. Anti-diabetic and hypoglycemic effects of Momordica charantia (bitter melon): A mini review. Br J Nutr. 2009;102: 1703–1708. doi: 10.1017/S0007114509992054 19825210 10.1017/S0007114509992054

[16] Porchezhian E, Dobriyal RM. An overview on the advances of Gymnema sylvestre: Chemistry, pharmacology and patents. Pharmazie. 2003;58: 5–12. 12622244

[17] Leach MJ. Gymnema sylvestre for diabetes mellitus: A systematic review. J Altern Complement Med. 2007;13: 977–983. doi: 10.1089/acm.2006.6387 18047444 10.1089/acm.2006.6387

[18] Al-Romaiyan A, Liu B, Asare-Anane H, Maity CR, Chatterjee SK, Koley N, et al A novel Gymnema sylvestre extract stimulates insulin secretion from human islets in vivo and in vitro. Phytother Res. 2010;24(9): 1370–1376. doi: 10.1002/ptr.3125 20812281 10.1002/ptr.3125

[19] Basch E, Ulbricht C, Kuo G, Szapary P, Smith M. Therapeutic applications of fenugreek. Altern Med Rev. 2003;8(1): 20–27. 12611558

[20] Neelakantan N, Narayanan M, de Souza RJ, van Dam RM. Effect of fenugreek (Trigonella foenum-graecum L.) intake on glycemia: a meta-analysis of clinical trials. Nutr J. 2014;13: 7 doi: 10.1186/1475-2891-13-7 24438170 10.1186/1475-2891-13-7 PMC3901758

[21] Kassaian N, Azadbakht L, Forghani B, Amini A. Effect of fenugreek seeds on blood glucose and lipid profiles in type 2 diabetic patients. Int J Vitam Nutr Res. 2009;79: 34–39. doi: 10.1024/0300-9831.79.1.34 19839001 10.1024/0300-9831.79.1.34

[22] Gupta A, Gupta R, Lal B. Effect of Trigonella foenum-graecum (fenugreek) seeds on glycaemic control and insulin resistance in type 2 diabetes mellitus: a double blind placebo controlled study. J Assoc Physicians India. 2001;49: 1057–1061. 11868855

[23] Arias BA, Ramón-Laca L. Pharmacological properties of citrus and their ancient and medieval uses in the Mediterranean region. J Ethnopharmacol. 2005;97(1): 89–95. doi: 10.1016/j.jep.2004.10.019 15652281 10.1016/j.jep.2004.10.019

[24] Karimi E, Oskoueian E, Hendra R, Oskoueian A, Jaafar HZ. Phenolic compounds characterization and biological activities of Citrus aurantium bloom. Molecules. 2012;17(2): 1203–1218. doi: 10.3390/molecules17021203 23442980 10.3390/molecules17021203 PMC6268598

[25] Jabri Karoui I, Marzouk B. Characterization of bioactive compounds in Tunisian bitter orange (Citrus aurantium L.) peel and juice and determination of their antioxidant activities. Biomed Res Int. 2013;2013: 345415 doi: 10.1155/2013/345415 23841062 10.1155/2013/345415 PMC3697287

[26] Haaz S, Fontaine KR, Cutter G, Limdi N, Perumean-Chaney S, Allison DB. Citrus aurantium and synephrine alkaloids in the treatment of overweight and obesity: an update. Obes Rev. 2006;7(1): 79–88. doi: 10.1111/j.1467-789X.2006.00195.x 16436104 10.1111/j.1467-789X.2006.00195.x

[27] Stohs SJ, Preuss HG, Shara M. A review of the human clinical studies involving Citrus aurantium (bitter orange) extract and its primary protoalkaloid p-synephrine. Int J Med Sci. 2012;9(7): 527–538. doi: 10.7150/ijms.4446 22991491 10.7150/ijms.4446 PMC3444973

[28] Armstrong WJ, Johnson P, Duhme S. The effect of commercial thermogenic weight loss supplement on body composition and energy expenditure in obese adults. JEP online. 2001;4(2): 28–34.

[29] Pellati F, Benvenuti S, Melegari M, Firenzuoli F. Determination of adrenergic agonists from extracts and herbal products of Citrus aurantium L. var. amara by LC. J Pharm Biomed Anal. 2002;29(6):1113–1119. 12110397 10.1016/s0731-7085(02)00153-x

[30] Rayalam S, Della-Fera MA, Baile CA. Phytochemicals and regulation of the adipocyte life cycle. J Nutr Biochem. 2008;19(11): 717–726. doi: 10.1016/j.jnutbio.2007.12.007 18495457 10.1016/j.jnutbio.2007.12.007

[31] Summers SA, Whiteman EL, Birnbaum MJ. Insulin signaling in the adipocyte. Int J Obes Relat Metab Disord. 2000;24 Suppl 4: S67–70. 11126246 10.1038/sj.ijo.0801509

[32] Tang QQ, Lane MD. Adipogenesis: from stem cell to adipocyte. Annu Rev Biochem. 2012;81: 715–36. doi: 10.1146/annurev-biochem-052110-115718 22463691 10.1146/annurev-biochem-052110-115718

[33] Jefcoate CR, Wang S, Liu X. Methods that resolve different contributions of clonal expansion to adipogenesis in 3T3-L1 and C3H10T1/2 cells. Methods Mol Biol. 2008;456: 173–193. doi: 10.1007/978-1-59745-245-8_13 18516561 10.1007/978-1-59745-245-8_13

[34] Green H, Kehinde O. An established preadipose cell line and its differentiation in culture. II. Factors affecting the adipose conversion. Cell. 1975;5(1): 19–27. 165899 10.1016/0092-8674(75)90087-2

[35] Trayhurn P, Beattie JH. Physiological role of adipose tissue: white adipose tissue as an endocrine and secretory organ. Proc Nutr Soc. 2001;60(3): 329–339. 11681807 10.1079/pns200194

[36] Coelho M, Oliveira T, Fernandes R. Biochemistry of adipose tissue: an endocrine organ. Arch Med Sci. 2013;9(2): 191–200. doi: 10.5114/aoms.2013.33181 23671428 10.5114/aoms.2013.33181 PMC3648822

[37] Rosen ED, Spiegelman BM. Adipocytes as regulators of energy balance and glucose homeostasis. Nature. 2006 12 14;444(7121):847–53. Review. doi: 10.1038/nature05483 17167472 10.1038/nature05483 PMC3212857

[38] Jabri Karoui I, Marzouk B. Characterization of bioactive compounds in Tunisian bitter orange (Citrus aurantium L.) peel and juice and determination of their antioxidant activities. Biomed Res Int. 2013;2013:345415 doi: 10.1155/2013/345415 23841062 10.1155/2013/345415 PMC3697287

[39] Longo M, Spinelli R, D’Esposito V, Zatterale F, Fiory F, Nigro C, et al Pathologic endoplasmic reticulum stress induced by glucotoxic insults inhibits adipocyte differentiation and induces an inflammatory phenotype. Biochim Biophys Acta. 2016;1863(6 PtA): 1146–1156. 26940722 10.1016/j.bbamcr.2016.02.019

[40] Raciti GA, Spinelli R, Desiderio A, Longo M, Parrillo L, Nigro C, et al Specific CpG hyper-methylation leads to Ankrd26 gene downregulation in white adipose tissue of a mouse model of diet-induced obesity. Sci Rep. 2017;7: 43526 doi: 10.1038/srep43526 28266632 10.1038/srep43526 PMC5339897

[41] Fiory F, Parrillo L, Raciti GA, Zatterale F, Nigro C, Mirra P, et al PED/PEA-15 inhibits hydrogen peroxide-induced apoptosis in Ins-1E pancreatic beta-cells via PLD-1. PLoS One. 2014;9(12): e113655 doi: 10.1371/journal.pone.0113655 25489735 10.1371/journal.pone.0113655 PMC4260953

[42] Song Y, Park HJ, Kang SN, Jang SH, Lee SJ, Ko YG, et al Blueberry peel extracts inhibit adipogenesis in 3T3-L1 cells and reduce high-fat diet-induced obesity. PLoS One. 2013;8(7):e69925 doi: 10.1371/journal.pone.0069925 23936120 10.1371/journal.pone.0069925 PMC3723699

[43] Liu XF, Bera TK, Kahue C, Escobar T, Fei Z, Raciti GA, et al ANKRD26 and its interacting partners TRIO, GPS2, HMMR and DIPA regulate adipogenesis in 3T3-L1 cells. PLoS One. 2012;7(5): e38130 doi: 10.1371/journal.pone.0038130 22666460 10.1371/journal.pone.0038130 PMC3364200

[44] Shi J, Kandror KV. Study of glucose uptake in adipose cells. Methods Mol Biol. 2008;456: 307–315. doi: 10.1007/978-1-59745-245-8_23 18516571 10.1007/978-1-59745-245-8_23

[45] Raciti GA, Iadicicco C, Ulianich L, Vind BF, Gaster M, Andreozzi F, et al Glucosamine-induced endoplasmic reticulum stress affects GLUT4 expression via activating transcription factor 6 in rat and human skeletal muscle cells. Diabetologia. 2010;53: 955–965. doi: 10.1007/s00125-010-1676-1 20165829 10.1007/s00125-010-1676-1

[46] Cassese A, Raciti GA, Fiory F, Nigro C, Ulianich L, Castanò I, et al Adenoviral gene transfer of PLD1-D4 enhances insulin sensitivity in mice by disrupting phospholipase D1 interaction with PED/PEA-15. PLoS One. 2013;8(4):e60555 doi: 10.1371/journal.pone.0060555 23585839 10.1371/journal.pone.0060555 PMC3621763

[47] Passaretti F, Tia M, D'Esposito V, De Pascale M, Del Corso M, Sepulveres R, et al Growth-promoting action and growth factor release by different platelet derivatives. Platelets. 2014;25(4):252–256. doi: 10.3109/09537104.2013.809060 23855408 10.3109/09537104.2013.809060

[48] Tang QQ, Otto TC, Lane MD. Mitotic clonal expansion: a synchronous process required for adipogenesis. Proc Natl Acad Sci U S A. 2003;100(1): 44–49. doi: 10.1073/pnas.0137044100 12502791 10.1073/pnas.0137044100 PMC140878

[49] Lane MD, Tang QQ, Jiang MS. Role of the CCAAT enhancer binding proteins(C/EBPs) in adipocyte differentiation. Biochem Biophys Res Commun. 1999;266(3): 677–683. doi: 10.1006/bbrc.1999.1885 10603305 10.1006/bbrc.1999.1885

[50] Guo L, Li X, Tang QQ. Transcriptional regulation of adipocyte differentiation: a central role for CCAAT/enhancer-binding protein (C/EBP) β. J Biol Chem. 2015;290(2): 755–761. doi: 10.1074/jbc.R114.619957 25451943 10.1074/jbc.R114.619957 PMC4294498

[51] Zhang JW, Tang QQ, Vinson C, Lane MD. Dominant-negative C/EBP disrupts mitotic clonal expansion and differentiation of 3T3-L1 preadipocytes. Proc Natl Acad Sci U S A. 2004;101(1): 43–47. doi: 10.1073/pnas.0307229101 14688407 10.1073/pnas.0307229101 PMC314135

[52] Tang QQ, Otto TC, Lane MD. CCAAT/enhancer-binding protein beta is required for mitotic clonal expansion during adipogenesis. Proc Natl Acad Sci U S A. 2003;100(3): 850–855. doi: 10.1073/pnas.0337434100 12525691 10.1073/pnas.0337434100 PMC298690

[53] Tang QQ, Lane MD. Activation and centromeric localization of CCAAT/enhancer-binding proteins during the mitotic clonal expansion of adipocyte differentiation. Genes Dev. 1999;13(17): 2231–2241. 10485846 10.1101/gad.13.17.2231 PMC316997

[54] Christy RJ, Kaestner KH, Geiman DE, Lane MD. CCAAT/enhancer binding protein gene promoter: binding of nuclear factors during differentiation of 3T3-L1 preadipocytes. Proc Natl Acad Sci U S A. 1991;88(6): 2593–2597. 2006196 10.1073/pnas.88.6.2593 PMC51279

[55] Zhang JW, Klemm DJ, Vinson C, Lane MD. Role of CREB in transcriptional regulation of CCAAT/enhancer-binding protein beta gene during adipogenesis. J Biol Chem. 2004;279(6):4471–4478. doi: 10.1074/jbc.M311327200 14593102 10.1074/jbc.M311327200

[56] Farmer SR. Transcriptional control of adipocyte formation. Cell Metab. 2006;4: 263–273. doi: 10.1016/j.cmet.2006.07.001 17011499 10.1016/j.cmet.2006.07.001 PMC1958996

[57] Wronkowitz N, Romacho T, Sell H, Eckel J. Adipose tissue dysfunction and inflammation in cardiovascular disease. Front Horm Res. 2014;43: 79–92. doi: 10.1159/000360560 24943300 10.1159/000360560

[58] Fajas L, Fruchart JC, Auwerx J. Transcriptional control of adipogenesis. Curr Opin Cell Biol. 1998;10: 165–173. 9561840 10.1016/s0955-0674(98)80138-5

[59] Rosen ED, Sarraf P, Troy AE, Bradwin G, Moore K, Milstone DS, et al PPAR gamma is required for the differentiation of adipose tissue in vivo and in vitro. Mol Cell. 1999;4: 611–617. 10549292 10.1016/s1097-2765(00)80211-7

[60] Weisiger RA. Cytosolic fatty acid binding proteins catalyze two distinct steps in intracellular transport of their ligands. Mol Cell Biochem. 2002;239(1–2): 35–43. 12479566

[61] Kim GS, Park HJ, Woo JH, Kim MK, Koh PO, Min W, et al Citrus aurantium flavonoids inhibit adipogenesis through the Akt signalling pathway in 3T3-L1 cells. BMC Complement Altern Med. 2012;12: 31 doi: 10.1186/1472-6882-12-31 22471389 10.1186/1472-6882-12-31 PMC3350436

[62] Kim HG, Kim GS, Park S, Lee JH, Seo ON, Lee SJ, et al Flavonoid profiling in three citrus varieties native to the Republic of Korea using liquid chromatography coupled with tandem mass spectrometry: contribution to overall antioxidant activity. Biomed Chromatogr. 2012;26(4):464–470. doi: 10.1002/bmc.1688 21830229 10.1002/bmc.1688

[63] Lee DH, Park KI, Park HS, Kang SR, Nagappan A, Kim JA, et al Flavonoids Isolated from Korea Citrus aurantium L. Induce G2/M Phase Arrest and Apoptosis in Human Gastric Cancer AGS Cells. Evid Based Complement Alternat Med. 2012;2012:515901 doi: 10.1155/2012/515901 22194772 10.1155/2012/515901 PMC3238396

[64] Mattoli L, Cangi F, Maidecchi A, Ghiara C, Tubaro M, Traldi P. A rapid liquid chromatography electrospray ionization mass spectrometry(n) method for evaluation of synephrine in Citrus aurantium L. samples. J Agric Food Chem. 2005 12 28;53(26):9860–6. doi: 10.1021/jf051270+ 16366666 10.1021/jf051270+

[65] Arbo MD, Larentis ER, Linck VM, Aboy AL, Pimentel AL, Henriques AT, et al Concentrations of p-synephrine in fruits and leaves of Citrus species (Rutaceae) and the acute toxicity testing of Citrus aurantium extract and p-synephrine. Food Chem Toxicol. 2008 8;46(8):2770–5. doi: 10.1016/j.fct.2008.04.037 18571300 10.1016/j.fct.2008.04.037

