# Supplementary material for: Citrus aurantium L. dry extracts promote C/ebpβ expression and improve adipocyte differentiation in 3T3-L1 cells
Source: PLoS One. 2018 Mar 29;13(3):e0193704. doi: 10.1371/journal.pone.0193704 (PMC5875749; doi:10.1371/journal.pone.0193704)
Supplement: S1 File — It includes Materials and Methods A, Table A, Table B, and Figure A in S1 File. Materials and Methods A in S1 File. UHPLC-PDA and FT-ICR-MS conditions, and identification and quantification of flavonoids. Table A in S1 File. Qualitative and quantitative flavonoid profile of CAde. Table B in S1 File. Effects of CAde on 3T3-L1 and NIH-3T3 cell cycle progression and cell proliferation. Figure A in S1 File. The effects of CAde on gene expression during adipogenesis in 3T3-L1 cells. (PDF) [file pone.0193704.s001.pdf]

S1 File. Supporting information file. It includes Materials and Methods A, Table A, Table B, and Figure A in S1 File.

Materials and Methods A in S1 File.

UHPLC-PDA and FT-ICR-MS conditions, and identification and quantification of flavonoids. UHPLC-PDA analyses were performed on a Shimadzu Nexera UHPLC system, consisting of a CBM-30A controller, two LC-30AD dual-plunger parallel-flow pumps, a DGU-20 A_{R5} degasser, a SPD-M30A photo diode array detector (equipped with a 1 µL detector flow cell volume), a CTO-30A column oven, and a SIL-30AC auto-sampler (Shimadzu Corp., Kyoto, Japan). The optimal mobile phase consisted of 0.1% HCOOH/H_2O *v/v* (A) and 0.1% HCOOH/ACN *v/v* (B). Analysis was performed in gradient elution as follows: 0.01-2.50 min, 5-15% B; 2.50-10.00 min, 15-25% B; 10.00-12.00 min, 25-55% B; 12.00-14.50 min, 55-65% B; 14.50-17.00, 65-70% B; 17.00-17.01, 70-5% B; 17.01-21.00, isocratic to 5% B. For UHPLC-MS/MS analyses a Kinetex^{TM} C18 150 mm × 2.1 mm (L.×I.D.), 2.6 µm column was employed (Phenomenex, Castel Maggiore, Italy). Flow rate was 0.5 mL/min. Column oven temperature was set to 40°C. Injection volume was 2 µL of extract. Data acquisition was set in the range 190-400 nm and chromatograms were monitored at 280 and 330 nm at the maximum absorbance of the compounds. The UHPLC system was coupled online to a FT-ICR Solarix 7T mass spectrometer through an ESI source (Bruker, Bremen, Germany). Resolution, sensitivity, and mass number calibration tuned using a standard sample solution of sodium trifluoroacetate. After the calibrant had flowed, cleaning operation of the tube and ESI probe was carried out by flowing acetonitrile (0.2 mL/min, 20 min). MS detection was operated both positive and negative ionization mode with the following parameters: drying gas temperature, 250°C; nebulizing gas flow (N_2), 2 L/min; drying gas pressure, 8 L/min. Full scan MS data were acquired in the range of 200-800 m/z, ion accumulation time, 150 ms. MS/MS experiments were conducted in data dependent acquisition, precursor ions were acquired in the range 150-800 m/z; ion accumulation time, 400 ms. LC-MS data elaboration was performed by the Bruker Data Analysis®. Identification

and quantification of flavonoids was carried out on the basis of standard retention time, UV spectra, and comparing molecular formulas generated by the analysis of accurate MS and MS/MS spectra with those present in literature. For quantification, 8 compounds were selected as external standards: diosmetin 6,8-di-C-glucoside, neohesperidin, eriocitrin, isoquercetin, narirutin, diosmetin, hesperetin and tangeretin. Stock solution (1 $mg.mL^{-1}$) were prepared in methanol. Calibration curves were obtained in a concentration range of 0.5-100 $\mu g.mL^{-1}$ with seven concentration levels. Peak areas of each standard were plotted against corresponding concentrations ($\mu g.mL^{-1}$). The amount of each compound in the sample was expressed as milligram per gram of extract. Linear regression was used to generate calibration curve, R^2 values were ≥ 0.995. The qualitative and quantitative flavonoid profiles of *CA*de are reported in the Table A.

Table A in S1 File. Qualitative and quantitative flavonoid profile of *CA*de

Peak	$[M-H]^-$	$[M-H]^+$	MS^2 *m/z*	Error (ppm)	Regression Curve	R^2	Extract flavonoids	Compound
1	593.1518	−	353.0691	1.18	$y = 1.2604E^{-06}\ x - 1.8347E^{-03}$	0.9981	55.56 ± 2.47	Vicenin-2
2	623.1628	−	383.0791	1.60	$y = 1.2604E^{-06}\ x - 1.8347E^{-03}$	0.9981	23.58 ± 2.55	Lucenin-2 4’-methyl ether
3	609.1478	−	301.0382	2.79	$y = 1.0012E^{-06}\ x - 2.2930E^{-03}$	0.9953	10.45 ± 2.32	Neohesperidin
4	595.1692	−	288.9530	4.03	$y = 1.2657E^{-06}\ x - 2.3642\ E^{-04}$	0.9962	3.06 ± 0.21	Eriocitrin
5	463.0890	−	301.0353	1.73	$y = 8.9337E^{-07}\ x - 1.7211E^{-03}$	0.9983	2.86 ± 0.37	Isoquercitrin
6	579.1686	−	271.0579	1.35	$y = 1.3943E^{-06}\ x - 2.4228E^{-03}$	0.9957	67.51 ± 1.98	Narirutin
7	607.1317	−	300.0320	1.98	$y = 4.7437E^{-07}\ x + 1.5354E^{-04}$	0.9996	3.01 ± 0.41	Neodiosmin
8	609.1786	−	301.0690	2.28	$y = 6.6021E^{-05}\ x - 1.15505E^{-03}$	0.9966	39.05 ± 1.94	Hesperidin
9	593.1833	−	285.0707	3.20	$y = 6.4061E^{-07}\ x - 1.9685E^{-03}$	0.9966	1.75 ± 0.19	Didymin
10	−	373.1236	343.0805	3.48	$y = 4.2086E^{-07}\ x + 9.9601E^{-04}$	0.9985	3.53 ± 1.14	Isosinensetin
11	−	403.1371	373.0918	-3.97	$y = 4.2086E^{-07}\ x + 9.9601E^{-04}$	0.9985	1.25 ± 0.57	Hexamethoxyflavone
12	−	373.1268	312.0990	3.49	$y = 4.2086E^{-07}\ x + 9.9601E^{-04}$	0.9985	8.57 ± 0.86	Sinensetin
13	−	403.1362	373.0923	-0.25	$y = 4.2086E^{-07}\ x + 9.9601E^{-04}$	0.9985	1.86 ± 0.54	Hexamethoxyflavone *isomer*
14	−	403.1329	373.0937	0.10	$y = 4.2086E^{-07}\ x + 9.9601E^{-04}$	0.9985	19.48 ± 0.88	Nobiletin
15	−	343.1166	282.0876	-2.91	$y = 4.2086E^{-07}\ x + 9.9601E^{-04}$	0.9985	3.77 ± 0.47	Tetramethyl-*o*-isoscutellarein
16	−	433.1472	403.1019	-4.85	$y = 4.2086E^{-07}\ x + 9.9601E^{-04}$	0.9985	3.96 ± 0.89	Heptamethoxyflavone
17	−	373.1268	343.0809	3.49	$y = 4.2086E^{-07}\ x + 9.9601E^{-04}$	0.9985	2.91 ± 0.49	Tangeretin

The amount of the compounds is expressed as milligram per gram of extract. Data are reported as mean ± relative standard deviation (RSD%) values of at least three independent experiments.

Table B in S1 File. Effects of *CA*de on 3T3-L1 and NIH-3T3 cell cycle progression and cell proliferation.

	**Ctrl**			***CA*de**		
**Cells**	**G1 (%)**	**S (%)**	**G2/M (%)**	**G1 (%)**	**S (%)**	**G2/M (%)**
**3T3-L1**	74.33 ± 0.11	8.10 ± 0.10	17.56 ± 0.05	74.20 ± 0.28	8.55 ± 0.64	17.25 ± 0.35
**NIH-3T3**	62.57 ± 1.86	11.97 ± 0.67	25.47 ± 1.76	63.05 ± 1.91	10.90 ± 0.85	26.05 ± 2.76
**3T3-L1 + MDI**	45.87 ± 2.61	37.83 ± 1.42	10.43 ± 2.34	29.00 ± 3.29***	49.10 ± 0.85###	16.00 ± 2.09¶
**NIH-3T3 + MDI**	76.93 ± 3.48	16.10 ± 1.39	6.97 ± 2.05	72.18 ± 3.70	19.53 ± 3.20	8.30 ± 0.62

	**Ctrl**			***CA*de**		
**Cells**	**24 h**	**48 h**	**72 h**	**24 h**	**48 h**	**72 h**
**3T3-L1 (x10^4)**	36.93 ± 3.16	48.75 ± 1.05***	62.33 ± 3.47***	38.00 ± 5.60	48.55 ± 1.95***	67.90 ± 5.20***
**NIH-3T3 (x10^4)**	52.90 ± 3.81	98.73 ± 5.66***	181.93 ± 16.48***	54.85 ± 2.41	95.26 ± 4.79***	202.26 ± 20.10***

Cell cycle progression of 3T3-L1 and NIH-3T3 cells was evaluated in untreated cells (Ctrl) or treated with *CA*de (100 µg/ml) for 16 h in presence or not of the adipogenic differentiation cocktail (MDI). The results are means ± SD of three independent experiments. Statistical analysis was performed using Student's t-test. ***p<0.001, *CA*de + MDI G1 Phase *vs.* Ctrl + G1 Phase; ###p<0.001, *CA*de + MDI S Phase *vs.* Ctrl + MDI S Phase; and ¶p<0.05, *CA*de + MDI G_2/M Phase *vs.* Ctrl + MDI G_2/M Phase.

Cell proliferation of 3T3-L1 and NIH-3T3 cells was evaluated in untreated cells (Ctrl) or treated with *CA*de (100 µg/ml) for 24, 48 and 72 h. The results are means ± SD of three independent experiments. Statistical analysis was performed using one-way ANOVA. ***p<0.001 *vs.* Ctrl at 24 h.

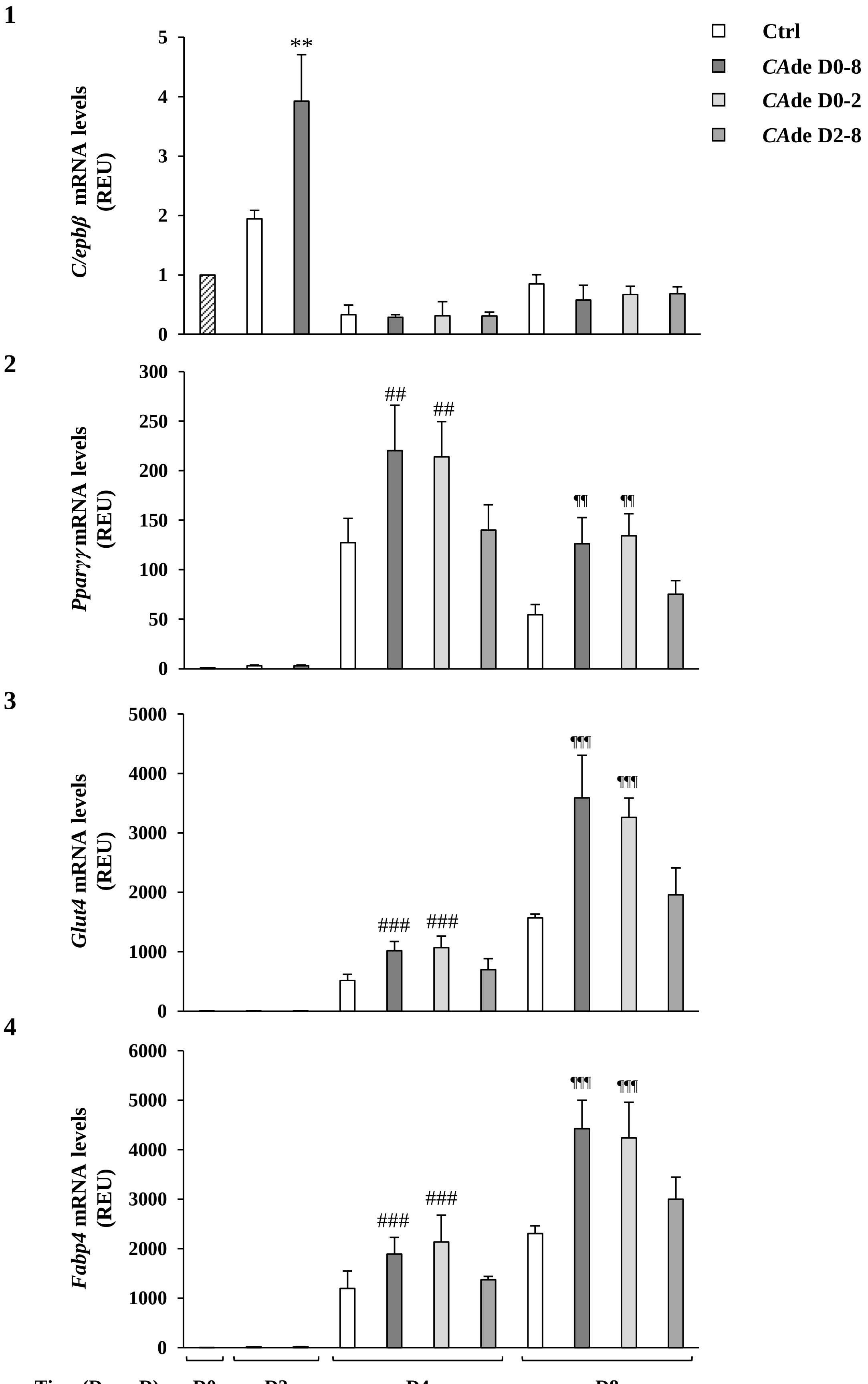

Figure A in S1 File. The effects of *CA*de on gene expression during adipogenesis in 3T3-L1 cells. 3T3-L1 pre-adipocytes were differentiated into mature adipocytes for 8 days in absence (Ctrl) or presence of *CA*de (100 µg/ml) from D0 to D2 (D0-D2), from D2 to D8 (D2-D8) and from D0 to D8 (D0-D8). qPCR was performed to detect the mRNA expression of (**1**) *C/ebpβ*, (**2**) *Pparγ*, and (**3**) *Glut4* and (**4**) *Fapb4* at D2, D4 and D8 upon adipogenic induction. Results are means ± SD of three independent experiments and were expressed as relative changes over control. Statistical analysis was performed using one-way ANOVA. $^{**}p<0.01$ *vs.* Ctrl at D2; $^{\#\#}p<0.01$ and $^{\#\#\#}p<0.001$ *vs.* Ctrl at D4; and $^{\P\P}p<0.01$ and $^{\P\P\P}p<0.001$ *vs.* Ctrl at D8.
